# Rescue of murine hind limb ischemia via angiogenesis and lymphangiogenesis promoted by cellular communication network factor 2

**DOI:** 10.1038/s41598-023-47485-y

**Published:** 2023-11-16

**Authors:** Masayuki Shimizu, Gumpei Yoshimatsu, Yuichi Morita, Tomoko Tanaka, Naoaki Sakata, Hideaki Tagashira, Hideichi Wada, Shohta Kodama

**Affiliations:** 1 Department of Cardiovascular Surgery, Faculty of Medicine, Fukuoka University, Fukuoka, Japan; 2 Department of Regenerative Medicine and Transplantation, Faculty of Medicine, Fukuoka University, 7-45-1 Nanakuma, Jonan-ku, Fukuoka, 814-0180 Japan; 3 Center for Regenerative Medicine, Fukuoka University Hospital, Fukuoka, Japan; 4 Department of Integrative Physiology, Graduate School of Medicine, Akita University, Akita, Japan

**Keywords:** Cardiology, Cell growth

## Abstract

Critical limb ischemia (CLI) is caused by severe arterial blockage with reduction of blood flow. The aim of this study was to determine whether therapeutic angiogenesis using cellular communication network factor 2 (CCN2) would be useful for treating CLI in an animal model. Recombinant CCN2 was administered intramuscularly to male C57BL/6J mice with hind limb ischemia. The therapeutic effect was evaluated by monitoring blood flow in the ischemic hind limb. In an in vivo assay, CCN2 restored blood flow in the ischemic hind limb by promoting both angiogenesis and lymphangiogenesis. VEGF-A and VEGF-C expression levels increased in the ischemic limb after treatment with CCN2. In an in vitro assay, CCN2 promoted proliferation of vascular and lymphatic endothelial cells, and it upregulated expression of *Tgfb1* followed by expression of *Vegfc* and *Vegfr3* in lymphatic endothelial cells under hypoxia. Suppression of *Tgfb1* did not affect the activity of CCN2, activation of the TGF-β/SMAD signaling pathway, or expression of *Vegfr3* in lymphatic endothelial cells. In summary, treatment using recombinant CCN2 could be a promising therapeutic strategy for CLI.

## Introduction

The prevalence of peripheral arterial disease (PAD) in the lower extremities has increased with rapid aging of the population^1^. Critical limb ischemia (CLI) is the most severe form of PAD and the strongest risk factor for ischemic rest pain, foot ulcers, and limb loss^2, 3^. The outcomes of surgical and endovascular interventions performed to recover arterial perfusion in the lower limbs in patients with CLI have been unsatisfactory. In a clinical study of patients with PAD in Canada, the 5-year mortality rate was 33.2%^4^. Furthermore, a multicenter randomized clinical trial in the UK found that bypass surgery or balloon angioplasty could not prevent amputation in half of all patients who underwent these procedures^5^. Therefore, there is a need for a radical and effective therapy that can rescue a limb from critical ischemia by restoring arterial flow.

Therapeutic angiogenesis using growth factors is garnering attention as a potential treatment for CLI^6, 7^. A number of animal studies have demonstrated the usefulness of therapeutic angiogenesis for vascularization using vascular endothelial growth factor (VEGF) in particular^8, 9^. Since early 2000s, clinical trials using growth factor have been promoted following the successes of these animal studies. For example, University of Minnesota group performed intracoronary infusion of recombinant human VEGF to 178 patients in angina, and revealed safety of this treatment with a partial improvement of quality of life in condition and frequency of angina^10^. Furthermore, Phase I/IIa clinical trial of therapeutic angiogenesis using hepatocyte growth factor gene transfer to 22 CLI patients was performed in Japan. It showed no adverse events correlated with this treatment for 6 months and approximately 70% of therapeutic effectiveness including treatment of ulcer^11^. Today, therapeutic angiogenesis using growth factor can be one of the promising treatments for CLI, and therefore, there is a need to identify a novel agent that can promote therapeutic angiogenesis and overcome CLI.

A potential treatment for CLI would be cellular communication network factor 2 (CCN2). CCN2, known as a connective tissue growth factor, is a cysteine-rich protein weighing 36–38 kDa^12^ and is expressed in the fibroblast, endothelial cells, chondrocytes, vascular smooth muscle cells, osteoblasts^13–16^. CCN2 has a number of physiological roles, including cell development, adhesion, proliferation, survival, and migration and production of extracellular matrix^17^. Furthermore, CCN2 has a role in promoting both angiogenesis^18^ and lymphangiogenesis^19^. These functions might contribute to recovery of blood flow in ischemic limb. We have previously demonstrated the effectiveness of angiogenesis and lymphangiogenesis in improving murine hind limb ischemia. In our opinion, lymphangiogenesis is also important to treat ischemic limb, because prevention of foot edema by promoting lymphatic drainage might contribute to recover the blood flow by removing interstitial pressure. Our previous study proved the importance of lymphangiogenesis in treating ischemic limb. For example, administration of vascular endothelial growth factor (VEGF)-C, a growth factor that promotes lymphangiogenesis, recovered the limb perfusion in ischemic limb^20^. Furthermore, xenotransplantation of porcine mesenchymal stem cells to relieve hind limb edema induced by promoting both angio-and lymphangiogenesis^21^. Therefore, we postulated that while its therapeutic effects in PAD are not fully elucidated^22, 23^, CCN2 could further improve the results of therapeutic angiogenesis for recovery of lower limb perfusion by promoting both angio- and lymphangiogenesis. The aims of this study were to determine whether CCN2 is an effective treatment for CLI and to identify the mechanism of its effect in a murine model of hind limb ischemia.

## Results

### Administration of CCN2 restored blood flow in ischemic hind limbs

**Figure 1:**
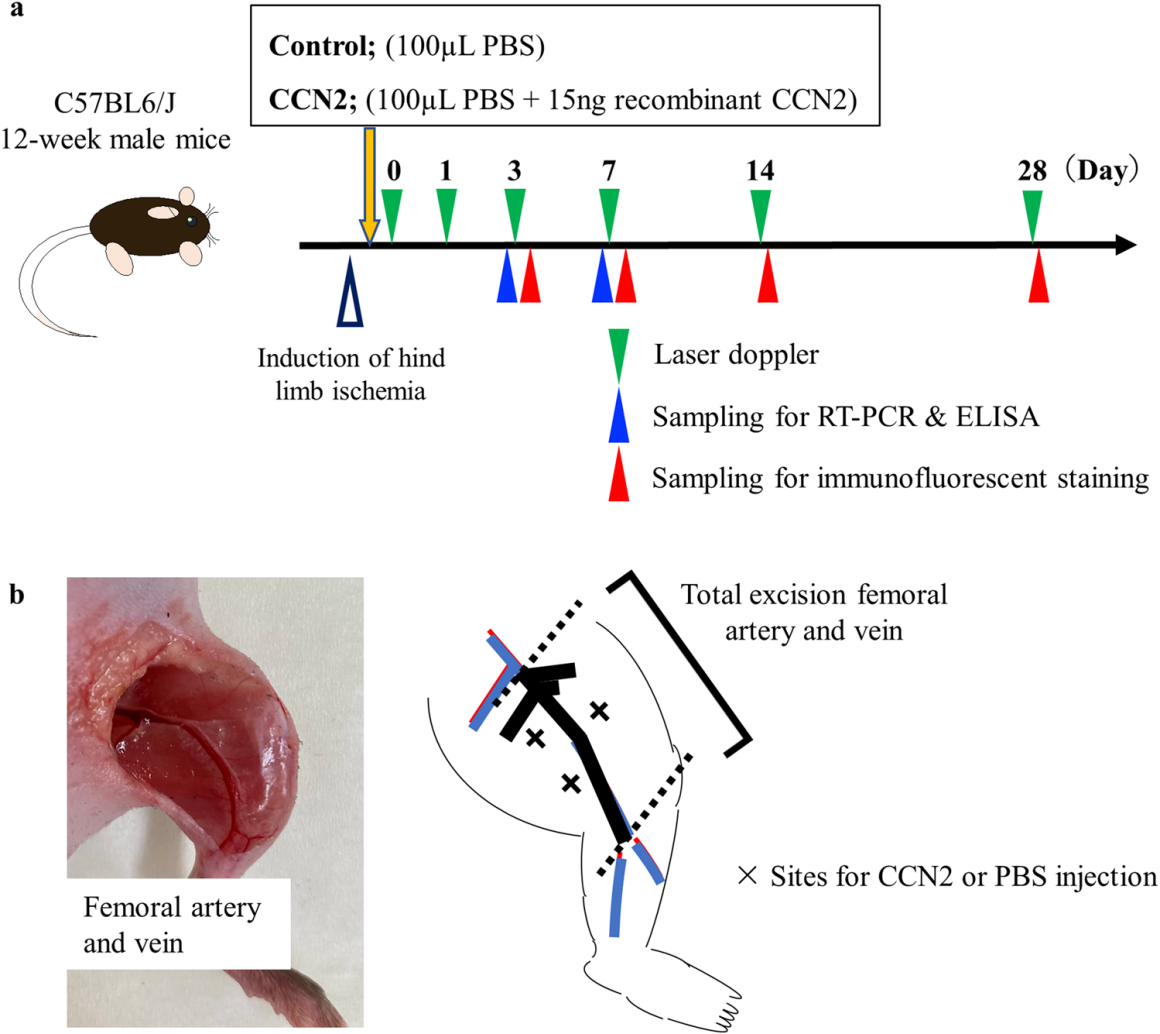
Study design. (**a**) Study design. Twelve-week-old male C57BL6/J mice with induced hind limb ischemia received an injection of CCN2 15 ng/PBS 100 μL (CCN2 group) or PBS 100 μL alone (control group). Blood flow in the hind limb was monitored by laser Doppler on postoperative days 0, 1, 3, 7, 14, and 28 (green triangles). Samples for RT-PCR, ELISA, and immunofluorescent staining were obtained from some mice on the scheduled days (blue and red triangles). (**b**) Diagram showing injection of CCN2 into the thigh muscles of mice with induced hind limb ischemia. *CCN2* cellular communication network factor 2, *ELISA* enzyme-linked immunosorbent assay, *PBS* phosphate-buffered saline, *RT-PCR* real-time polymerase chain reaction.

The control group received a percutaneous injection of 100 µL of PBS into the thigh muscle of the ischemic hindlimb at three points on the day of induction of ischemia (Fig. 1a,b). The CCN2 group received the PBS injection with 15 ng of recombinant rat CCN2 protein. Blood flow was monitored on postoperative days (PODs) 0, 1, 3, 7, 14, and 28. Hind limb samples were acquired on PODs 3, 7, 14, and 28 from some of the mice in each group for histological assessment and on PODs 3 and 7 for evaluation of genes and proteins.

**Figure 2:**
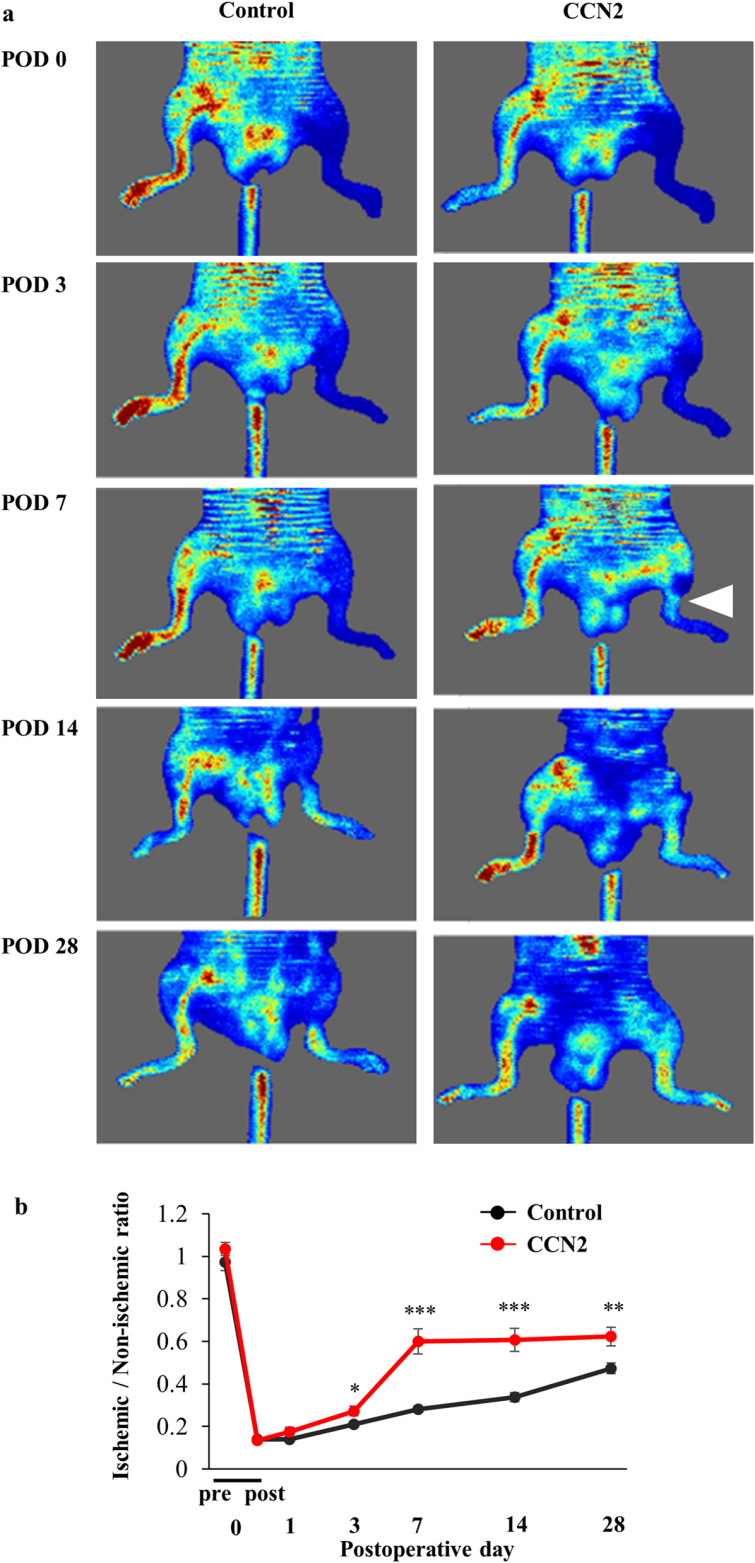
Blood flow in ischemic hind limbs after treatment with CCN2. (**a**) Representative laser Doppler images of mice in the control and CCN2 groups on postoperative days 0, 1, 3, 7, 14, and 28. Blood flow is visualized and differentiated by the colors red (rich blood flow) and blue (poor blood flow). The white triangle in the figure on postoperative day 7 in the CCN2 group indicates recovery of blood flow. (**b**) Change in the ischemic/non-ischemic ratio in the control group (n = 10) and the CCN2 group (n = 11). The data are expressed as the mean ± standard error. *P < 0.05; **P < 0.01; ***P < 0.001. *CCN2* cellular communication network factor 2, *POD* postoperative day.

The appropriate dose of CCN2 was determined by a preliminary examination in which mice received intramuscular injections of CCN2 into the ischemic hind limb and were then classified into four groups by dose (control and 15, 150, and 1500 ng). Blood flow was restored after POD 7 in mice that received 15 ng of CCN2 but not in the other three study groups (control, CCN2 150 ng, and CCN2 1500 ng) (Supplemental Fig. 1). Therefore, 15 ng was selected as the appropriate dose of CCN2 for this study. Figure 2a shows representative laser Doppler images of an ischemic hind limb in the control group and the CCN2 group. Restoration of blood flow was observed in both groups over time, with additional improvement in blood flow in the CCN2 group on POD 7. Blood flow in the ischemic hind limb was detected from the thigh to the ankle on POD 7 in the CCN2 group but not in the control group. However, blood flow in the hind limb was detected on POD 14 in the control group. The extent of blood flow was relatively prominent in the CCN2 group through to POD 28 (Fig. 2a). The ischemic/non-ischemic ratio was significantly higher in the CCN2 group than in the control group after POD 3 (Fig. 2b).

### CCN2 promoted both angiogenesis and lymphangiogenesis in the ischemic hind limb

**Figure 3:**
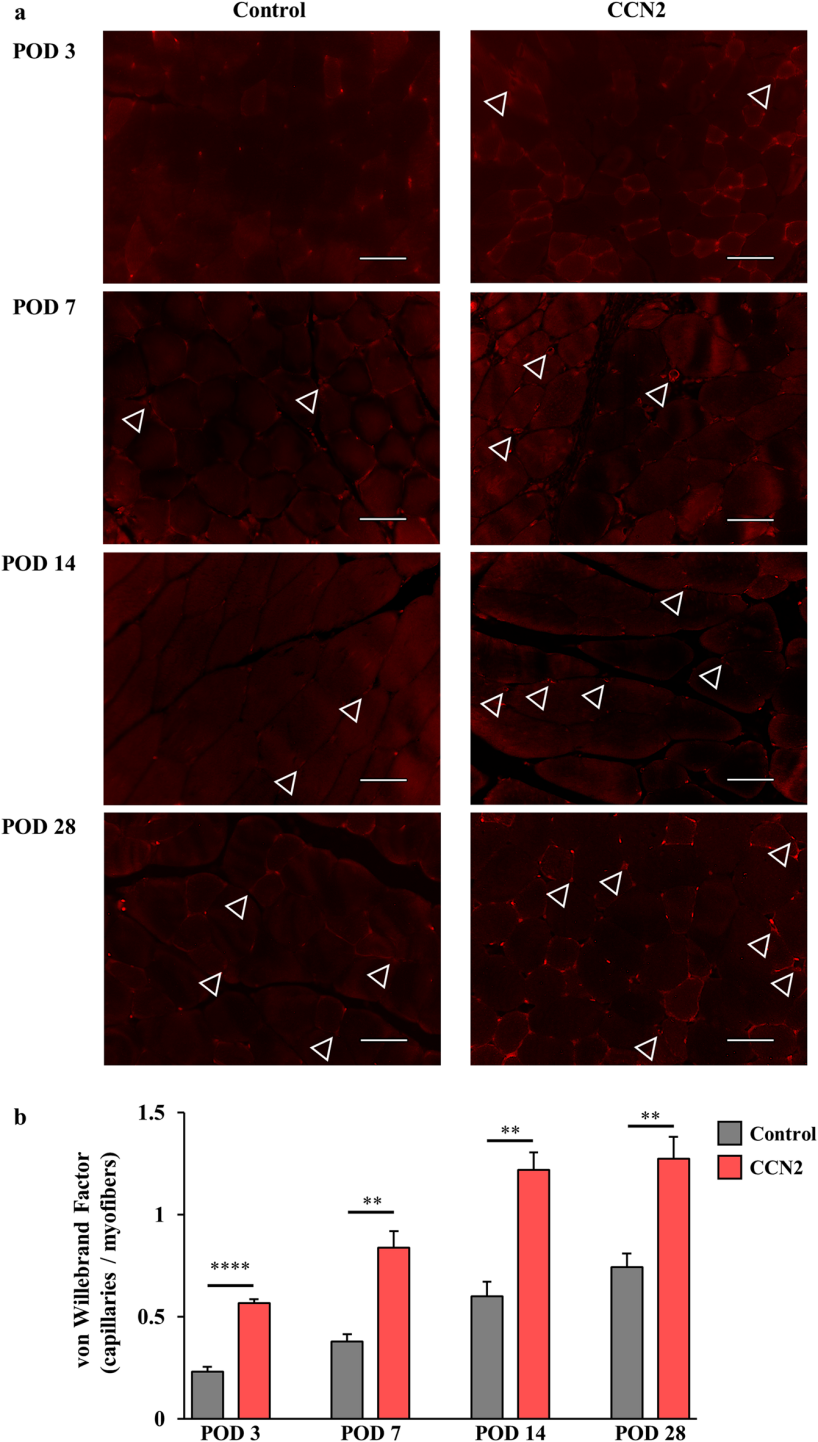
Blood vessels in the ischemic hind limb after treatment with CCN2. (**a**) von Willebrand factor (vWF)-positive capillaries (blood vessels) in myofibers of the ischemic hind limb in the control group (left) and the CCN2 group (right). The capillaries (stained in red) are indicated by white triangles. Original magnification, × 400; scale bar, 50 µm. (**b**) Change in density of vWF-positive capillaries per myofiber in the control group (n = 5; gray) and the CCN2 group (n = 5; light blue). The data are expressed as the mean ± standard error. *P < 0.05; **P < 0.01; ***P < 0.001; ****P < 0.0001. *CCN2* cellular communication network factor 2, *POD* postoperative day.

Figure 3a shows the vWF-positive blood vessels in the thigh muscles of the ischemic hind limbs in both groups. More blood vessels were seen in the CCN2 group than in the control group during the observation period (Fig. 3a). Furthermore, the density of vWF-positive capillaries (i.e., blood vessels) per myofiber was significantly higher in the CCN2 group (Fig. 3b).

**Figure 4:**
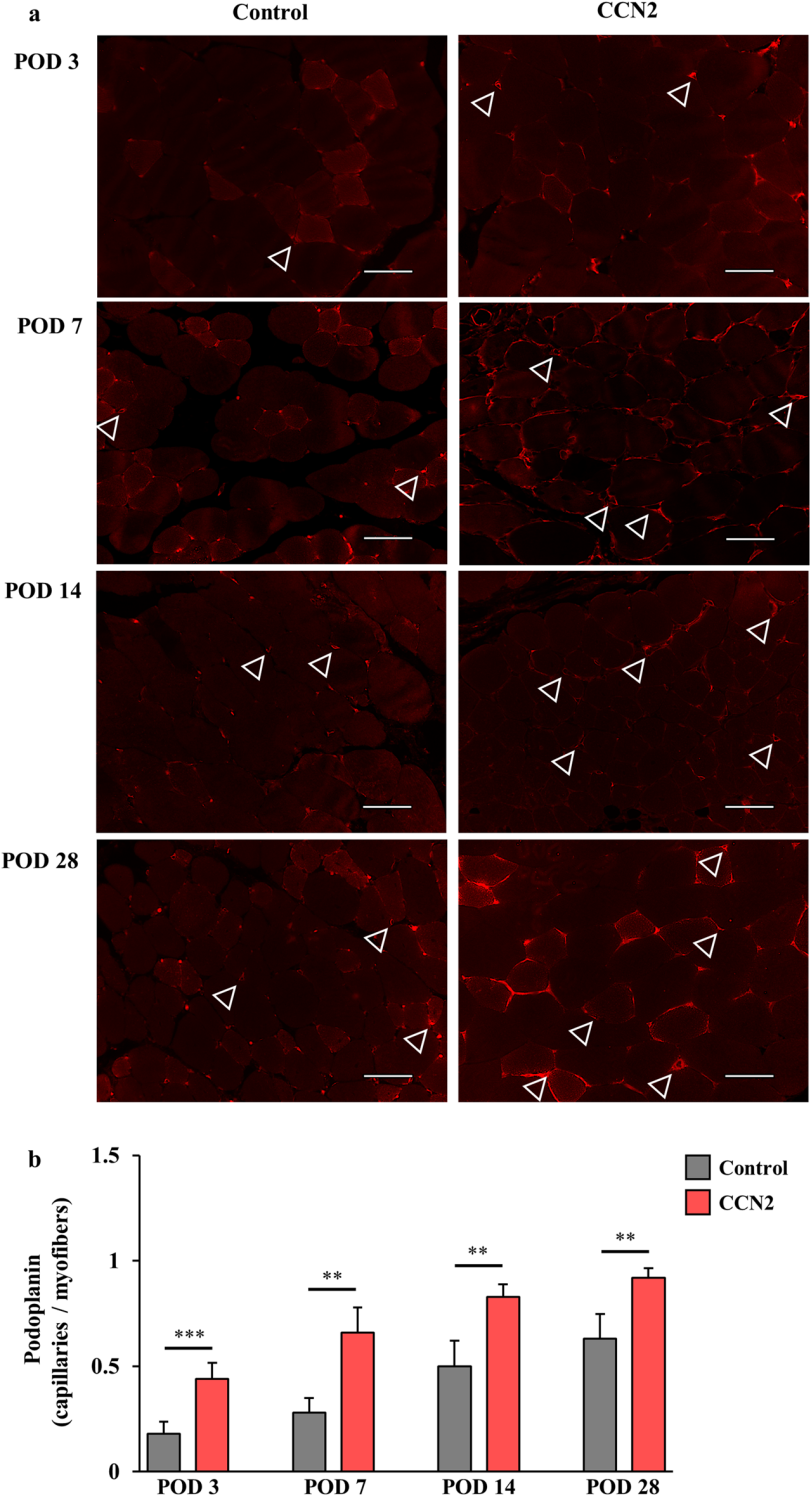
Lymphatic vessels in the ischemic hind limb after treatment with CCN2. (**a**) Podoplanin-positive capillaries (lymphatic vessels) in myofibers of the ischemic hind limb in the control group (left) and the CCN2 group (right). The capillaries (stained in red) are indicated by white triangles. Original magnification, × 400; scale bar, 50 µm. (**b**) Change in density of podoplanin-positive capillaries per myofiber in the control group (n = 5; gray) and the CCN2 group (n = 5; light blue). The data are expressed as the mean ± standard error. *P < 0.05; **P < 0.01; ***P < 0.001; ****P < 0.0001. *CCN2* cellular communication network factor 2, *POD* postoperative day.

As with angiogenesis, the number of podoplanin-positive capillaries indicating lymphatic vessels was significantly greater in the CCN2 group than in the control group at each assessment time (Fig. 4a). The density of podoplanin-positive capillaries per myofiber also increased with time and was significantly higher in the CCN2 group (Fig. 4b).

These results indicated that CCN2 can upregulate both angiogenesis and lymphangiogenesis and promote recovery of blood flow in an ischemic hind limb.

### CCN2 promoted expression of both VEGF-A and VEGF-C in the ischemic hind limb

**Figure 5:**
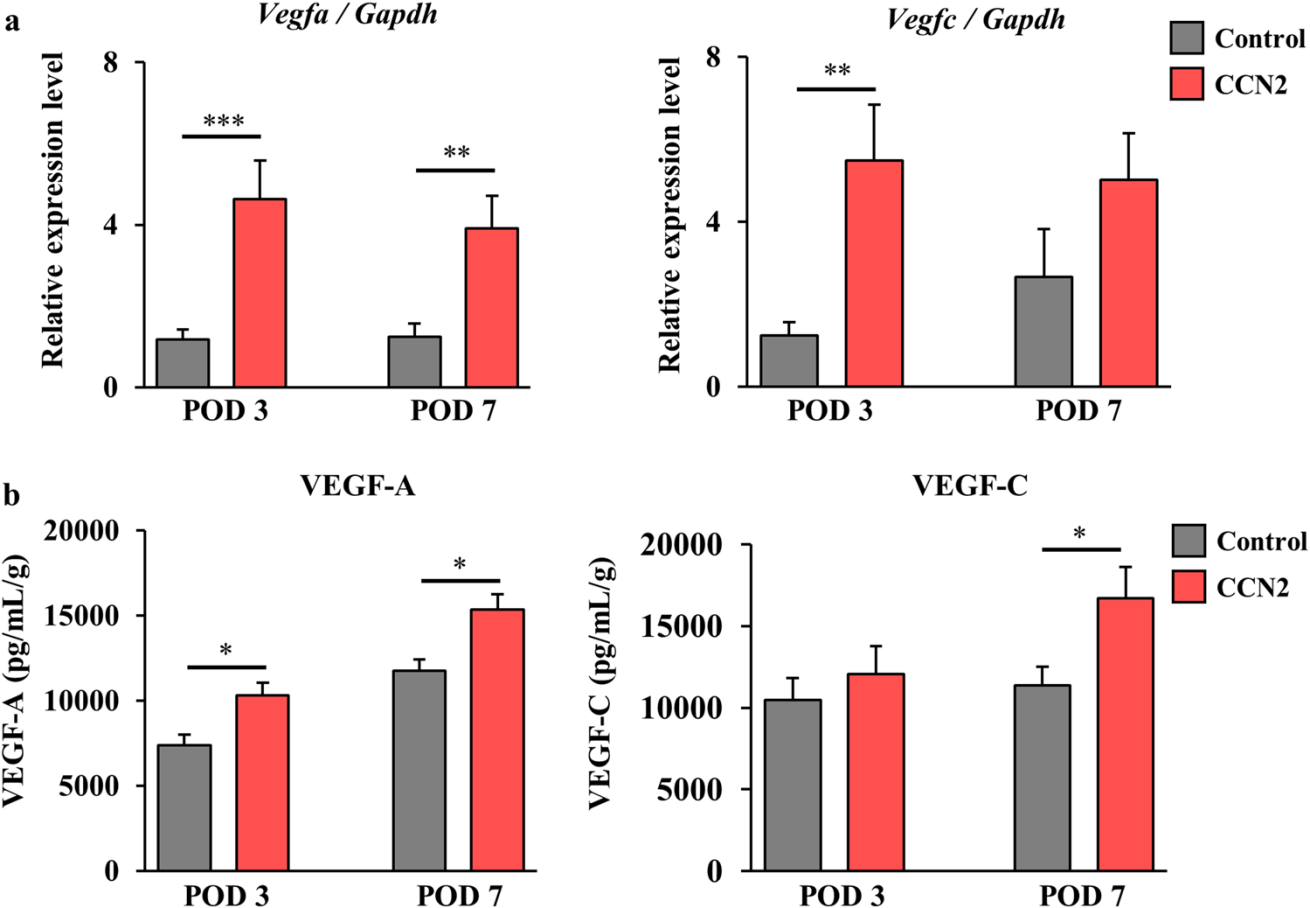
Gene expression and protein levels of angiogenesis and lymphangiogenesis markers in the thigh muscles after treatment with CCN2. (**a**) *Vegfa* (left) and *Vegfc* (right) expression levels in the ischemic hind limb on postoperative days 3 and 7 in the control group (gray) and the CCN2 group (light blue), n = 7–8 per group. (**b**) VEGF-A (left) and VEGF-C (right) levels in the ischemic hind limb on postoperative days 3 and 7 in the control group (gray) and the CCN2 group (light blue), n = 6–7 per group. The data are expressed as the mean ± standard error, n = 6–7 per group. *P < 0.05; **P < 0.01; ***P < 0.001. *ns* not significant, *CCN2* cellular communication network factor 2, *POD* postoperative day, *VEGF* vascular endothelial growth factor.

Next, we examined the expression levels of growth factors that promote recovery of blood flow in the ischemic hind limb after administration of CCN2. Expression of the gene (*Vegfa*) for VEGF-A, a key mediator of angiogenesis, in tissue was significantly higher in the CCN2 group than in the control group on POD 3 (P < 0.01) and POD 7 (P < 0.01) (Fig. 5a, left panel). Expression of the gene (*Vegfc*) for VEGF-A, a growth factor contributing to lymphangiogenesis, was also upregulated in the CCN2 group on PODs 3 and 7. However, a significant difference in expression level was seen only on POD 3 (P < 0.01) (Fig. 5a, right panel). Expression levels of both *Vegfa* and *Vegfc* were highest on POD 3 in the CCN2 group.

Early overexpression of the *Vegfa* and *Vegfc* genes induced by administration of CCN2 promoted production of the corresponding proteins. Significantly greater production of VEGF-A protein was detected on POD 3 (P < 0.05) and POD 7 (P < 0.01) in the CCN2 group, reflecting increased expression of *Vegfa* expressions (Fig. 5b, left panel). A significant increase in production of VEGF-C protein in the CCN2 group was noted on POD 7 (P < 0.05) following overexpression of *Vegfc* on POD 3 (not significant) (Fig. 5b, right panel). These findings indicated that administration of CCN2 induced expression of *Vegfa* and *Vegfc* genes in the ischemic hind limb and subsequent production of VEGF-A, which contributes to angiogenesis, and of VEGF-C, which contributes to lymphangiogenesis.

### CCN2 promoted proliferation of both VECs and LECs under hypoxia

Next, we evaluated the influence of hypoxia on the effects of CCN2 in our model of ischemia. Although we found that CCN2 was useful for promoting angiogenesis and lymphangiogenesis and for restoration of blood flow, it was unclear whether and how hypoxia affected the usefulness of CCN2.

**Figure 6:**
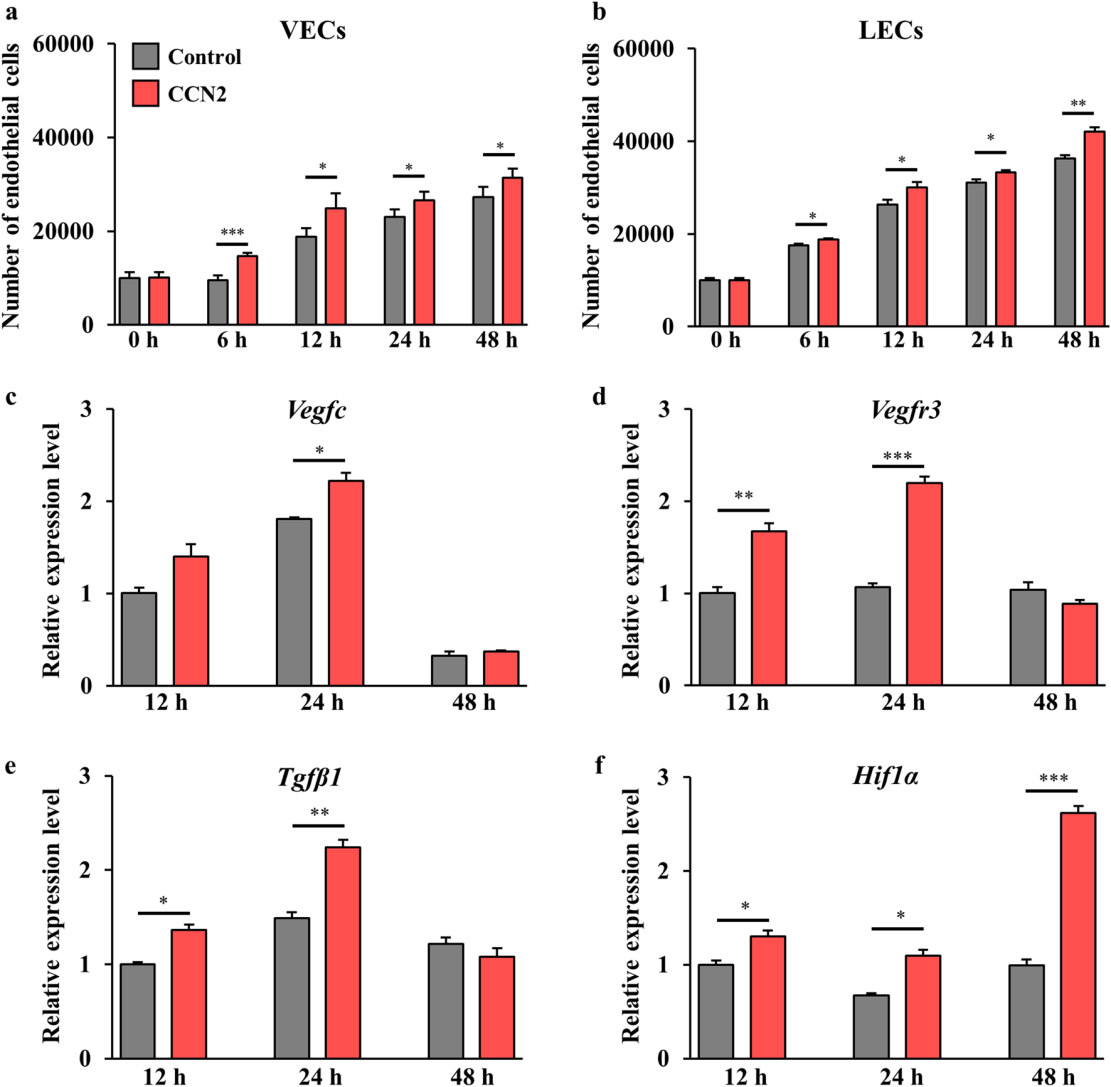
Effects of CCN2 on proliferation of VECs and LECs and lymphangiogenesis-correlated gene expression under hypoxia. The number of murine VECs [passage 6, n = 5; (**a**)] and LECs [passage 6, n = 5; (**b**)] cultured with and without CCN2 (CCN2 group and control group, respectively) under hypoxia after 0, 6, 12, 24, and 48 h of incubation. Cellular proliferation was assessed by the MTS assay. The data are expressed as the mean ± standard error. *P < 0.05; **P < 0.01; ***P < 0.001. *ns* not significant. (**c**–**f**) Changes in expression of *Vegfc* (**c**), *Vegfr3* (**d**), *Tgfβ1* (**e**), and *Hif1α* (**f**) in LECs cultured with and without CCN2 (CCN2 group and control group, respectively) under hypoxia. *Gapdh* was used as an internal control, and gene expression levels were expressed relative to *Gapdh* mRNA. The data are expressed as the mean ± standard error, n = 3/group. *P < 0.05; **P < 0.01; ***P < 0.001. *CCN2* cellular communication network factor 2, *LECs* lymphatic endothelial cells, *VECs* vascular endothelial cells.

First, we assessed the contribution of CCN2 to proliferation of both VECs and LECs under normoxic and hypoxic conditions. Figure 6a,b shows that both VECs and LECs proliferated in a time-dependent manner under hypoxia in both the control group and the CCN2 group and that cell proliferation was significantly greater in the CCN2 group. However, cell proliferation was not prominent in either of the study groups under normoxia (Supplemental Fig. 2a,b). These findings indicated that hypoxia might enhance the therapeutic effects of CCN2.

### CCN2 enhanced induction of lymphangiogenic genes in LECs under hypoxia

We then assessed the contribution of CCN2 to expression of genes associated with angiogenesis and lymphangiogenesis in VECs and LECs under hypoxia. Figure 6c–e shows the relative expression levels of *Vegfc, Vegfr3* (the gene for the VEGF-C receptor), and *Tgfβ1* in LECs after 12, 24 and 48 h under hypoxia. Expression levels of *Vegfc*, *Vegfr3* and *Tgfβ1* were significantly higher by 24 h in the CCN2 group. Notably, expression of these genes peaked at 24 h. Expression of *Vegfa* in VECs under hypoxia was significantly higher in the CCN2 group at 24 h but not at 12 or 48 h (Supplemental Fig. 3a). However, *Vegfc* was significantly upregulated in the CCN2 group at each time point (Supplemental Fig. 3b). These data suggested that administration of recombinant CCN2 in a hypoxic state promotes not only early expression of genes corresponding to the VEGF family but also activation of the TGF-β signaling pathway in VECs and LECs.

Next, we assessed the effect of administration of CCN2 on expression of the *Hypoxia inducible factor 1 subunit alpha* (*Hif1a*) gene in LECs. HIF-1α is a transcription factor that is induced during a state of hypoxia and promotes vascularization and angiogenesis via activation of VEGF. Our data indicated that expression of *Hif1α* was significantly upregulated in the CCN2 group at each time point under hypoxia (Fig. 6f).

### Angiogenic and lymphangiogenic effects of CCN2 under hypoxia depended on the TGF-β/SMAD signaling pathway

**Figure 7:**
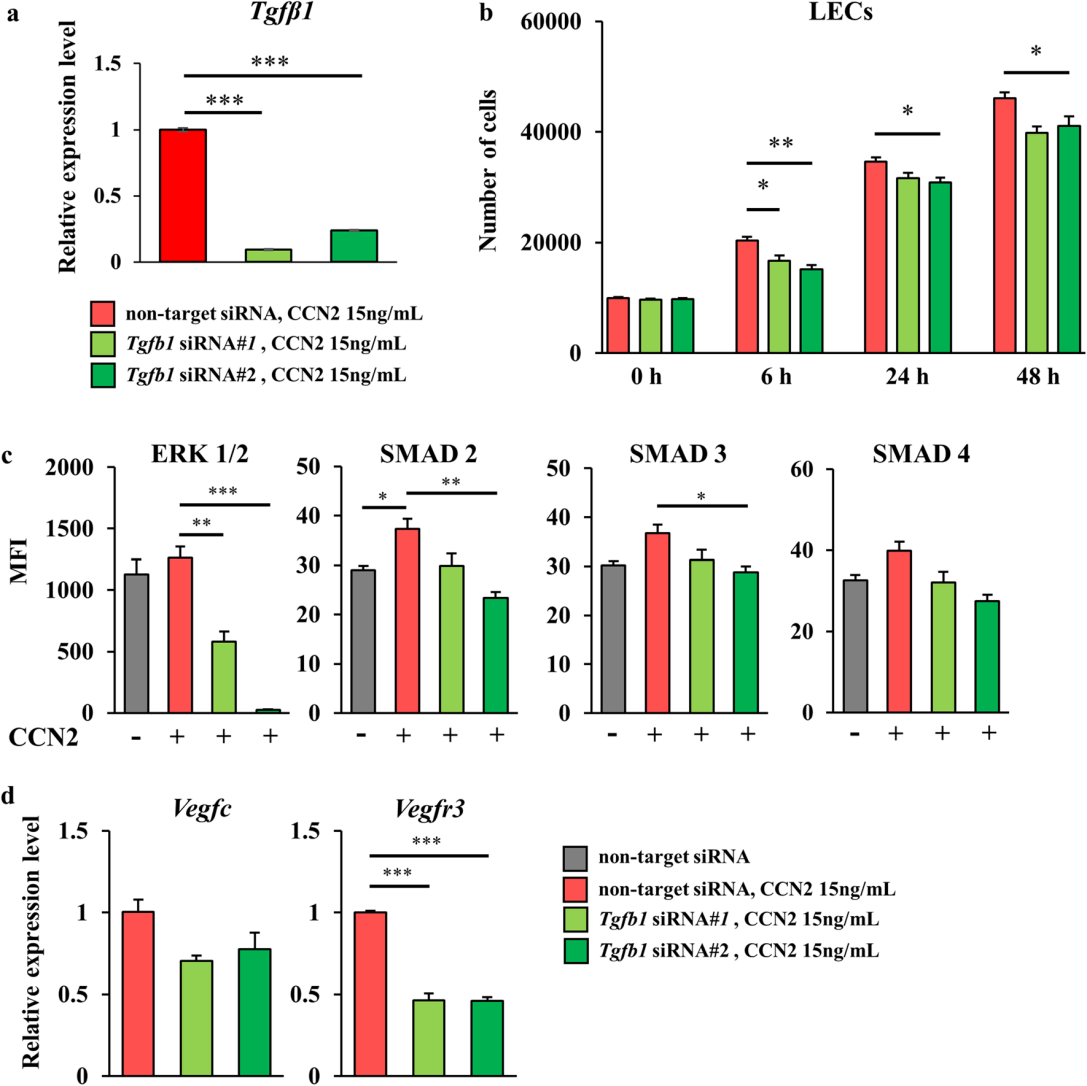
Influence of *Tgfβ1* knockdown on lymphangiogenesis in LECs under hypoxia. *Tgfb1* knockdown were performed by transfection with siRNA against *Tgfb1* using two types of *Tgfb1* siRNA (siRNA#1 and siRNA#2). (**a**) Expression level of *Tgfβ1 i*n LECs after administration of CCN2 under hypoxia. The data are expressed as the mean ± standard error, n = 3/group. (**b**) Cellular proliferation of LECs after administration of CCN2 under hypoxia. The data are expressed as the mean ± standard error. The LECs were in passage 6, n = 5/group. (**c**) Levels of ERK 1/2, SMAD 2, SMAD 3, and SMAD 4 in LECs after administration of CCN2 under hypoxia. The median fluorescence intensity of these proteins was measured using the Luminex^®^ system, n = 3/group. (**d**) Expression levels of *Vegfc* and *Vegfr3* in LECs after administration of CCN2 under hypoxia. The data are expressed as the mean ± standard error, n = 3/group. *P < 0.05; **P < 0.01; ***P < 0.001. *ns* not significant, *CCN2* cellular communication network factor 2, *ERK* extracellular signal-regulated kinase, *LECs* lymphatic endothelial cells, *SMAD* suppressor of mothers against decapentaplegic.

It is known that TGF-β is a potent inducer of CCN2 transcription^24^. Moreover, Valle-Tenney et al. showed that hypoxia and TGF-β1 induced expression of CCN2 synergistically in skeletal muscle^25^. Therefore, we postulated that TGF-β was a key factor in the angiogenic and lymphangiogenic effects of CCN2 under hypoxia. Assessment of the therapeutic effects of CCN2 using TGF-β1 knockdown LECs confirmed that CCN2-induced proliferation of LECs under hypoxia was canceled by knockdown of TGF-β1 (Fig. 7a,b).

We then investigated the expression of downstream signals in the TGF-β signaling pathway, including ERK 1/2 and SMAD 2, 3, and 4, by administration of CCN2 under hypoxia using the Multiplex Magpix immunoassay. We detected an increase in phospho-SMAD 2 in response to administration of CCN2 and decreases in the phosphorylation levels of ERK 1/2, SMAD 2, and SMAD 3 by knockdown of *Tgfb1* (Fig. 7c). *Tgfb1* knockdown also suppressed the CCN2-induced increase in expression of *Vegfr3* under hypoxia (Fig. 7d). These findings suggested that the angiogenic and lymphangiogenic effects of CCN2 under hypoxia depended on the TGF-β/SMAD signaling pathway.

## Discussion

Identification of a radical and curative therapy for CLI is a pivotal challenge. Therapeutic angiogenesis is a candidate treatment that has the potential to dramatically improve the prognosis and quality of life of patients with CLI. However, although a large number of animal studies have indicated that this therapeutic strategy can promote angiogenesis, the evidence for its benefit in clinical trials has been equivocal. For example, the first trial of therapeutic angiogenesis using recombinant VEGF showed no significant benefit in patients with angina^10^. In another clinical trial, intracoronary infusion of fibroblast growth factor-2 did not improve cardiac function in patients with coronary artery disease but reduced symptoms of angina^26^. It is possible that the limited efficacy of the therapeutic angiogenesis approach reflects the growth factors used. VEGF is a typical growth factor used for therapeutic angiogenesis but has the disadvantage of hyperpermeability in newly formed vessels^27^. Vascular hyperpermeability allows leaking of interstitial fluid and proteins, which worsens edema, such that therapeutic angiogenesis is unable to achieve adequate blood flow^28^. We consider that rich vascularization, promotion of venous return, and lymphatic drainage are essential attributes of a growth factor used for therapeutic angiogenesis.

In this study, we evaluated the ability of CCN2 to restore blood flow by promotion of angiogenesis and lymphangiogenesis in an animal model of CLI. We revealed that CCN2 harbored similar therapeutic effectiveness in recovery of blood flow in ischemic hind limb to our previous studies. For example, significant increase of the blood flow was seen on day 7 by CCN2 and VEGF-C^20^. On the other hand, xenotransplantation of porcine MSCs significantly improved the blood flow on day 3^21^. Promotion of these two processes by CCN2 was confirmed by hyperexpression of *Vegfa* and *Vegfc* followed by overproduction of VEGF-A and VEGF-C in VECs and LECs in ischemic hind limbs. We also confirmed that promotion of these growth factors is required for activation of the TGF-β/SMAD signaling pathway, which contributes to growth and proliferation of VECs and LECs and possibly to both angiogenesis and lymphangiogenesis. Furthermore, suppression of the TGF-β/SMAD signaling pathway prevented expression of *Vegfr3* in LECs, indicating that CCN2 also promoted lymphangiogenesis by increasing expression of VEGF receptor-3 via activation of the TGF-β/SMAD signaling pathway. It is generally recognized that CCN2 is a downstream mediator of TGF-β signaling^29, 30^ and that binding of CCN2 to TGF-β promotes a positive feedback loop that increases TGF-β signaling^31, 32^. Furthermore, the therapeutic effects of CCN2 were enhanced under hypoxia, which reflects the microenvironment in hind limb ischemia. Interestingly, our data confirm that administration of CCN2 increases the expression of *Hif1α* in LECs (and probably also in VECs), which might contribute to neovascularization via activation of VEGF. Previous studies have also shown that CCN2 regulates the expression of VEGF and TGF-β1 via expression of HIF-1α under hypoxia^22, 33^. These data indicate that therapeutic angiogenesis using CCN2 could be a promising treatment for CLI.

As discussing above, we focused on TGF-β signaling pathway for elucidating angio- and lymphangiogenic functions in this study. On the other hand, we also consider that other signaling pathway except TGF-β might contribute to activation of CCN2, because proliferation of LECs was not absolutely inhibited by cancellation of *Tgfb*, while expression of downstream target (ex. ERK1/2) was prohibited. As other candidate pathway, we speculate that Hippo signaling pathway plays a role in cellular proliferation and anti-apoptotic function via CCN2. Hippo signaling pathway and the co-transcription factor Yes-associated protein (YAP) signaling pathway are important regulators for development, organ growth, homeostasis, and cancer by modulating cell proliferation, differentiation, and apoptosis^34, 35^. For example, dysregulation of Hippo signaling pathway induces chronic pancreatitis via CCN2 upregulation^36^. Hippo signaling pathway is also associated with anti-metastasis function via inhibition of CCN2^37^. Therefore, proliferation of LECs under hypoxia might also induced by regulation of Hippo signaling pathway.

While our study revealed the usefulness of CCN2 in angio- and lymphangiogenesis, the duration of lasting recombinant CCN2 after injection was unclear. We estimated that intramuscular injected rat CCN2 might be eliminated within a couple of hours, following the findings in previous publication, Gerritsen KGF, et al. performed intravenous injection of recombinant human CCN2 into mice. They revealed that the CCN2 was eliminated within an hour by hepatic metabolism^38^. We consider intramuscular injected rat CCN2 might be lasted at least an hour because of avoiding hepatic metabolism. In other words, CCN2 could contribute to angiogenesis in a short period of the presence.

Supplemental Fig. 1 showed CCN2 played angiogenic role at 15 ng, but the role was canceled at 150 and 1500 ng. In general, many biological molecules harbor biological functions in a dose dependent manner, but some, including CCN2, do not. At lower doses, CCN2 might work as a trigger of specific signaling pathways or cellular responses that are conducive to angiogenesis and blood flow restoration. However, at higher doses, CCN2 might activate different or even inhibitory pathways, leading to a less favorable outcome.

In this study, we certified newly formed vessels by CCN2 administration was immature without basement membrane and pericytes. This might be because angio- and lymphangiogenesis by CCN2 were occurred via induction. VEGF. It is considered that the characteristics of vessels are similar between induction of CCN2 and VEGF. Therefore, further modification which induce maturation might be necessary for neovascularization using CCN2.

While the usefulness of CCN2 in treating hind limb ischemia and the mechanism of its therapeutic effects have been partially elucidated, the mechanism is still not understood in detail. The interpretation for the mechanisms of detailed function of CCN2 was disrupted by the complicated phenomenon induced by the signaling pathway, such as Notch, TLR and HIF-1, associated with ischemia^39–41^. For example, it is unclear why and how administration of CCN2 increases the expression of *Hif1a*, how it regulates the TGF-β/SMAD signaling pathway in terms of angiogenesis and lymphangiogenesis, and whether CCN2 promotes angiogenesis and lymphangiogenesis via expression of VEGF or promotion of HIF1α. However, we anticipate that further elucidation of the physiological characteristics of CCN2 will contribute to development of this growth factor for therapeutic angiogenesis in CLI in the future.

In conclusion, we have demonstrated that CCN2 promotes both angiogenesis and lymphangiogenesis in CLI. Focusing on CCN2 may help to develop a novel strategy for the treatment of CLI.

## Methods

### Animal model of hind limb ischemia

The ischemic hind limb model was constructed using 12-week-old C57BL/6J mice (Charles River Laboratories Japan, Inc., Yokohama, Japan). The care of the mice and experimental procedures followed the “Principles of Laboratory Animal Care” (Guide for the Care and Use of Laboratory Animals, 8th edition, National Research Council, 2011). The experimental protocol was approved by the Animal Care and Use Committee of Fukuoka University (approval number: 2009044), and this study was reported in accordance with ARRIVE guidelines 2.0 (https://arriveguidelines.org/arrive-guidelines). Hind limb ischemia was induced as previously described^42, 43^. Briefly, the left femoral artery and vein were exposed and resected from the proximal portion near the inguinal ligament to the distal portion of the saphenous artery. The remaining arterial branches were also excised. The contralateral hind limb served as an internal control. All surgeries were performed under general anesthesia using isoflurane (Fujifilm Wako Pure Chemical Corporation, Osaka, Japan).

### Study groups and experimental design

The mice were randomly allocated to a control group or a CCN2 group. The control group received a percutaneous injection of 100 µL of phosphate-buffered saline (PBS) into the thigh muscle of the ischemic hindlimb at three points using a 27-gauge needle on the day of induction of ischemia. The injection points were determined by equally divided three parts from the knee to inguinal ligament as same as the previous study^20^. The CCN2 group received the same treatment but with addition of 15 ng of recombinant rat CCN2 protein (Carrier Free; R&D Systems, Minneapolis, MN, USA) to the 100 µL of PBS injected. In the previous study, we elucidated the angiogenic function of CCN2 by infusion of recombinant rat CCN2 with Matrigel into back of mice^44^. Blood flow was monitored on postoperative days (PODs) 0, 1, 3, 7, 14, and 28. Hind limb samples were acquired on PODs 3, 7, 14, and 28 from some of the mice in each group for histological assessment and on PODs 3 and 7 for evaluation of genes and proteins.

### Measurement of blood flow in the hind limbs

Blood flow in the left (ischemic) and right (non-ischemic) hindlimbs was measured by laser Doppler perfusion (Moor Instruments Ltd, Devon, UK). The average of three measurements was used to calculate the ratio of blood flow between the ischemic and non-ischemic sides (ischemic/non-ischemic ratio) on each day point.

### Histological assessments

The thigh adductor muscles were obtained from some mice in each group for histological assessment. The samples were fixed in 10% formaldehyde solution and embedded in paraffin. Each specimen was cut to a thickness of 5 μm. Blood and lymphatic vessels were detected by immunofluorescent staining. The primary antibodies used were sheep polyclonal anti-von Willebrand factor (vWF, 1:100; Abcam, Cambridge, UK)^45^ for detecting blood vessels and purified hamster anti-podoplanin (1:100; Biolegend, San Diego, CA, USA)^46^ for detecting lymphatic vessels, collagen IV (information) for detecting basement membrane, and CD34 (information) for detecting pericytes. The secondary antibodies used were CyTM3-conjugated F(ab')2 fragment donkey anti-sheep IgG (H + L) antibody (1:100; Jackson Immunoresearch, Philadelphia, PA, USA) and goat anti-hamster IgG (H + L) Alexa Fluor 546 (1:100; Invitrogen; Thermo Fisher Scientific, Inc., Waltham, MA, USA). Histological images were obtained under a BZ-X700 microscope (Keyence Co., Osaka, Japan). The vWF/podoplanin positive cells were defined as vascular/lymphatic endothelial cells, when structured lumens with vWF/podoplanin staining cells were explicitly seen. The numbers of vWF-positive and podoplanin-positive capillaries per myofiber were counted and averaged by randomly selecting five fields (both groups with relatively large numbers) from three different regions (upper, middle, and lower thigh muscles) of each specimen in the control group and CCN2 group.

### Cell culture and proliferation assay

Primary vascular endothelial cells (VECs) and lymphatic endothelial cells (LECs) derived from C57BL/6J mice (Cell Biologics, Chicago, IL, USA) were maintained using an Endothelial Cell Growth Medium-2 Bullet Kit (Lonza, Basel, Switzerland) and used at passage 6.

For the proliferation assay, LECs and VECs were cultured using the Endothelial Cell Growth Medium-2 Bullet Kit supplemented with 5% fetal bovine serum at 37 °C under normoxic (21% O_2_) and hypoxic (5% O_2_) conditions. Cell proliferation capability was assessed by MTS assay (Promega, Madison, WI, USA). Specifically, 1 × 10^4^ viable cells per well were seeded with or without CCN2 in 96-well plates. The basal culture medium was changed to medium containing 15 ng/mL of recombinant rat CCN2 protein. At 0, 6, 24, and 48 h after treatment, 20 μL of MTS solution (Promega) were added to each well, and the cells were incubated for 2 h. The absorbance values for each well were measured at OD 490 nm using a Spark 10 M plate reader (Tecan, Mannedorf, Switzerland).

### Real-time polymerase chain reaction assay

VECs and LECs obtained from murine thigh muscle samples in both groups were lysed using TRIzol reagent (Invitrogen). RNA was extracted from the lysates using a PureLink RNA Mini kit (Thermo Fisher Scientific). The concentration of extracted RNA was measured using a NanoDrop 2000 spectrophotometer (Thermo Fisher Scientific). Complementary DNA was synthesized from total RNA using a PrimeScript^®^ PLUS RT Kit (Takara Biotechnology Co., Ltd., Kusatsu, Japan). Reverse transcription and real-time polymerase chain reaction (PCR) with the SYBR green assay was performed using the StepOnePlus™ Real-Time PCR System (Applied Biosystems; Thermo Fisher Scientific). Information about the primers used for PCR is provided in Supplemental Table 1. Relative quantification analysis was performed with StepOne software version 2.3 (Applied Biosystems). The results were normalized to *Gapdh* as a housekeeping gene. All reactions were performed in triplicate, and quantification was performed using the 2^−ΔΔCT^ method.

### Enzyme-linked immunosorbent assay

The thigh muscles were homogenized in T-PER Reagent (Thermo Fisher Scientific) with protease inhibitor cocktail (Roche, Mannheim, Germany). Next, the supernatant was collected after centrifugation at 10,000 rpm for 5 min. VEGF-A and VEGF-C protein levels in the thigh muscle lysates were determined by enzyme-linked immunosorbent assays (R&D Systems and Life Science Inc., St. Petersburg, FL, USA, respectively)^47^.

### Multiplex magnetic bead-based immunoassays

Multiplex magnetic bead-based immunoassays were performed using the Luminex Magpix^®^ system (Millipore, Billerica, MA, USA)^48^. Milliplex^®^ map kits (TGF-β Signaling Magnetic Bead Panel 6-plex (6-plex kit), Millipore) were used to detect targets in the transforming growth factor (TGF)-β signaling pathways. Murine LECs were lysed in Milliplex MAP Lysis Buffer (Millipore) with protease inhibitors (Nakalai Tesque, Kyoto, Japan). The protein concentration was determined using a BCA protein assay kit (Pierce, Thermo Fisher Scientific). Milliplex MAP assay buffer 2 (Millipore) was used to prepare the microsphere working mixture, and protein samples (1–25 µg, 1:20 dilution) were incubated at 4 °C overnight. After washing, the samples were incubated with detection antibody (1:20; Biotin, Millipore) at room temperature for 1 h. Next, the samples were incubated with the secondary antibody (1:25; Streptavidin–Phycoerythrin, Millipore) for 15 min after washing. The beads were then resuspended in Milliplex MAP assay buffer 2 and read using the Magpix system. Antibody responses were expressed as median fluorescence intensity per sample. Phosphorylated extracellular signal-regulated kinase (ERK) 1/2 and suppressor of mothers against decapentaplegic (SMAD) 2, 3, and 4 were monitored for changes.

### Gene silencing by siRNA

*Tgfb1* in LECs was suppressed by transfection with *Tgfb1* siRNA (Thermo Fisher Scientific). LECs (2.5 × 10^5^) were transfected with 50 pmol of *Tgfb1* siRNA or non-target siRNA (Thermo Fisher Scientific) using Lipofectamine RNAiMAX (Thermo Fisher Scientific) according to the manufacturer’s instructions. The following siRNA sequences for each mRNA target were used. *Tgfb1*#1: sense sequence 5′‐AACUCCACGUGGAAAUCAAtt‐3′, anti‐sense sequence 5′‐UUGAUUUCCACGUGGAGUUtg‐3′; *Tgfb1*#2: sense sequence 5′‐AAUCAAGUGUGGAGCAACAtt‐3′, antisense sequence 5′‐UGUUGCUCCACACUUGAUUtt‐3′; nonsilencing siRNA: Silencer™ Select Negative Control No. 1 siRNA (Thermo Fisher Scientific, 4390843).

### Statistical analysis

The data were examined for statistical significance using the Tukey–Kramer test for multiple comparison (ex. assessment of the blood flow among the different doses of CCN2 administration), or the Student’s *t*-test for comparison between two groups. All experiments were performed at least three times, and the data are expressed as the mean ± standard error. All analyses were performed using JMP 14.0 software (SAS Institute Inc, Cary, NC, USA). P-values < 0.05 were considered statistically significant.

### Supplementary Information

Supplementary Table 1.

Supplementary Figure 1.

Supplementary Figure 2.

Supplementary Figure 3.

## Data Availability

The datasets generated and analyzed during the current study are available in the “figshare” repository (10.6084/m9.figshare.23304704).

## Abbreviations

ANOVA: Analyses of variance
BCA: Bicinchoninic acid
CCN2: Cellular communication network factor 2
CLI: Critical limb ischemia
ELISA: Enzyme-linked immunosorbent assay
ERK: Extracellular signal-regulated kinase
FGF: Fibroblast growth factor
Gapdh: Glyceraldehyde-3-phosphate dehydrogenase
HIF-1α: Hypoxia-inducible factor-1 alpha
HRP: Horseradish peroxidase
LEC: Lymphatic endothelial cell
MBA: Multiplex magnetic bead-based immunoassay
MFI: Median fluorescence intensity
PAD: Peripheral arterial disease
PBS: Phosphate-buffered saline
PCR: Polymerase chain reaction
POD: Postoperative day
siRNA: Small interfering RNA
SMAD: Suppressor of mothers against decapentaplegic
TGF-β: Transforming growth factor-β
VEC: Vascular endothelial cell
VEGF: Vascular endothelial growth factor
vWF: Von Willebrand factor
