# Supplementary material for: Rescue of murine hind limb ischemia via angiogenesis and lymphangiogenesis promoted by cellular communication network factor 2
Source: Sci Rep. 2023 Nov 16;13:20029. doi: 10.1038/s41598-023-47485-y (PMC10654495; doi:10.1038/s41598-023-47485-y)
Supplement: Supplementary file 1 — Supplementary Table 1. [file 41598_2023_47485_MOESM1_ESM.docx]

**Supplemental Table 1. Information of primers**

| Primer name | Sequence (5′–3′) | Tm (°C) | Comments |
| --- | --- | --- | --- |
| *Gapdh_*F | TGTGTCCGTCGTGGATCTGA | 63.8 | Internal control |
| *Gapdh_*R | TTGCTGTTGAAGTCGCAGGAG | 63.9 |  |
| *Vegfa_*F | ACATTGGCTCACTTCCAGAAACAC | 63.8 | Angiogenic factor |
| *Vegfa_*R | TGGTTGGAACCGGCATCTTTA | 64.7 |  |
| *Vegfc_*F | CAGCAACATTACCACAGTGTCAG | 64.4 | Lymphangiogenic factor |
| *Vegfc_*R | TAGACATGCACCGGCAGGAA | 65.0 |  |
| *Flt4(Vegfr3)_*F | CTATGGCTGAGCCCAATGAC | 64.5 | Lymphangiogenic factor |
| *Flt4(Vegfr3)_*R | ACCTTATCAAAGATGCTCTCGG | 64.0 |  |
| *Tgfb1_*F | GTGTGGAGCAACATGTGGAACTCTA | 64.7 | Inducible factor of Vegfc expression |
| *Tgfb1_*R | CGCTGAATCGAAAGCCCTGTA | 64.7 |  |
| *Hif1a_*F | GCGTGCATGTCTAATCTGTTCC | 61.9 | Inducible factor of Vegfc expression |
| *Hif1a_*R | GATTCTGACATGCCACATAGCTC | 61.0 |  |
