# Supplementary figures and images for: Rescue of murine hind limb ischemia via angiogenesis and lymphangiogenesis promoted by cellular communication network factor 2
Source: Sci Rep. 2023 Nov 16;13:20029. doi: 10.1038/s41598-023-47485-y (PMC10654495; doi:10.1038/s41598-023-47485-y)

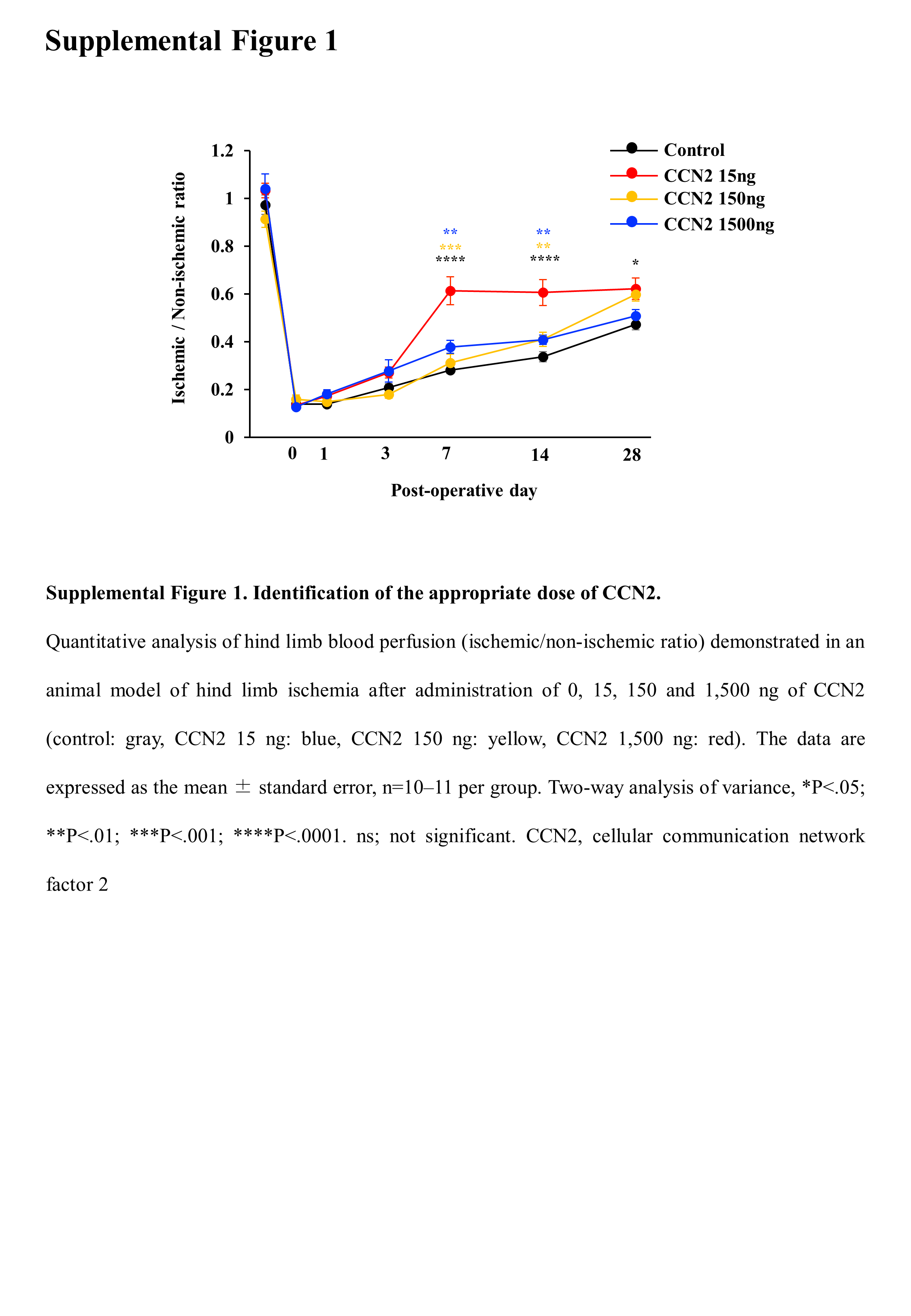

Supplement: Supplementary file 2 — Supplementary Figure 1. [file 41598_2023_47485_MOESM2_ESM.tif]

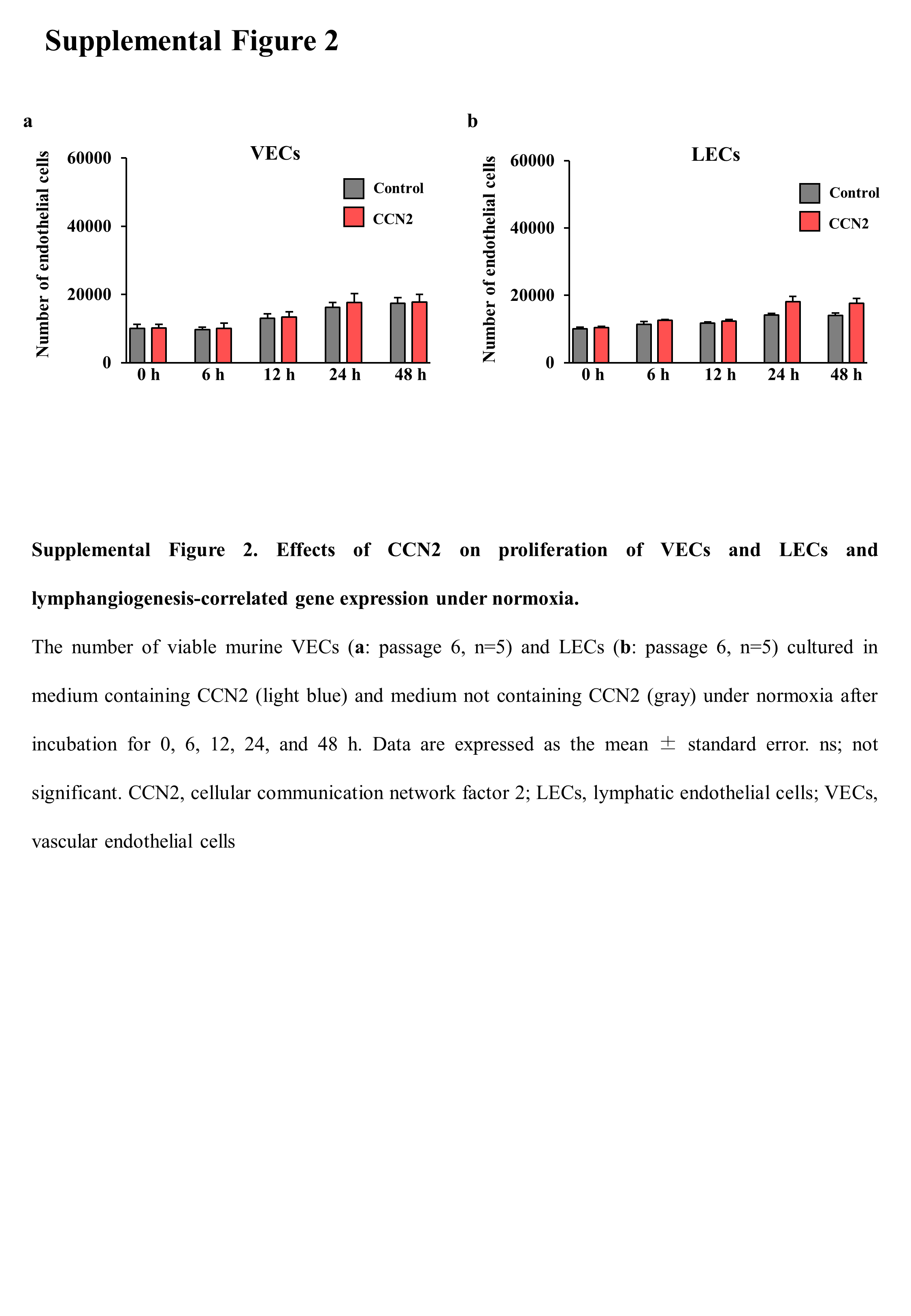

Supplement: Supplementary file 3 — Supplementary Figure 2. [file 41598_2023_47485_MOESM3_ESM.tif]

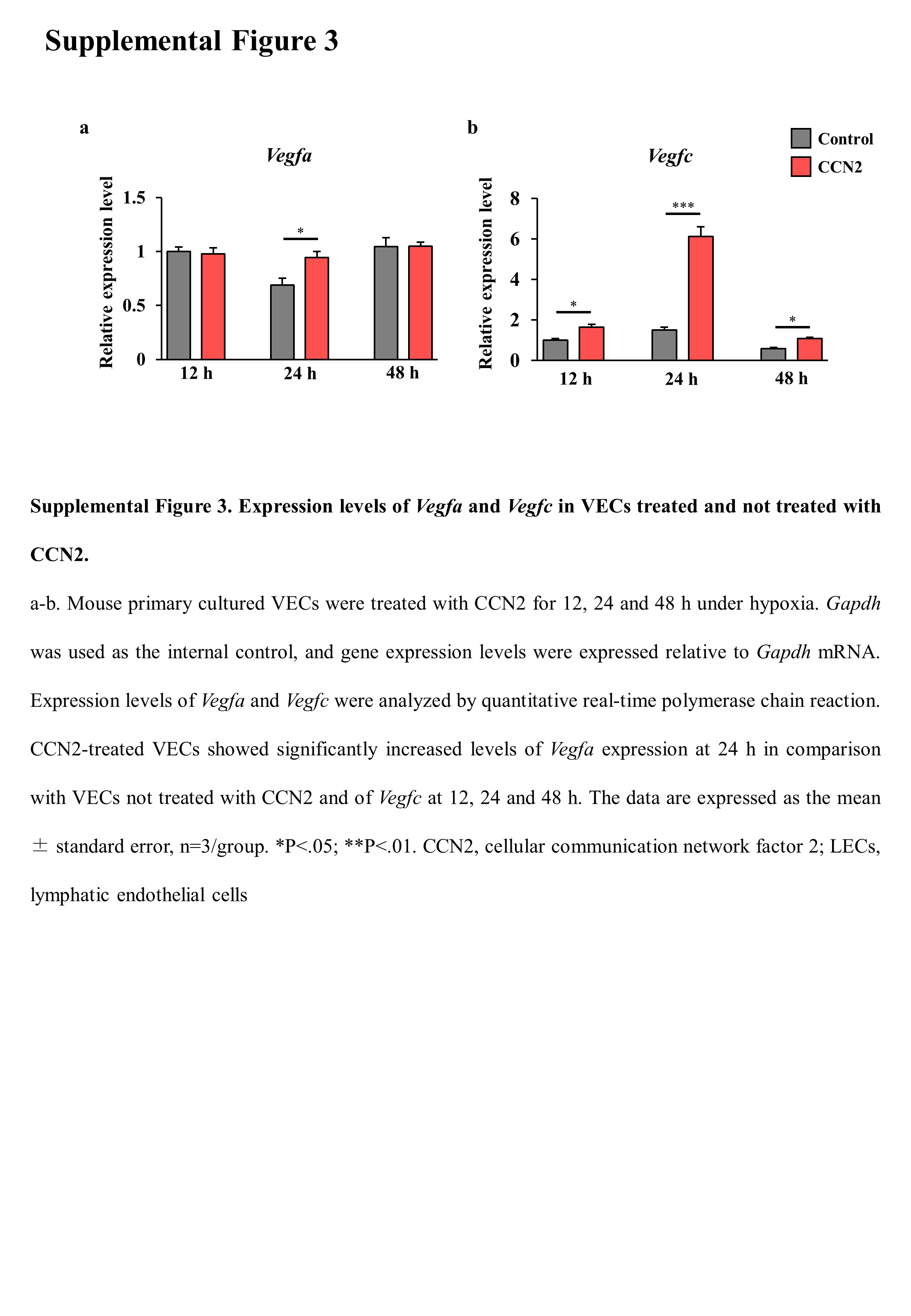

Supplement: Supplementary file 4 — Supplementary Figure 3. [file 41598_2023_47485_MOESM4_ESM.tif]
